# Looking Ahead: Health Impact Assessment of Front-of-Pack Nutrition Labelling Schema as a Public Health Measure

**DOI:** 10.3390/ijerph18041422

**Published:** 2021-02-03

**Authors:** Rodrigo Feteira-Santos, Violeta Alarcão, Osvaldo Santos, Ana Virgolino, João Fernandes, Carlota Pacheco Vieira, Maria João Gregório, Paulo Nogueira, Andreia Costa, Pedro Graça

**Affiliations:** 1 Instituto de Saúde Ambiental (ISAMB), Faculdade de Medicina, Universidade de Lisboa, Avenida Professor Egas Moniz, 1649-028 Lisboa, Portugal; rodrigosantos@medicina.ulisboa.pt (R.F.-S.); osantos@medicina.ulisboa.pt (O.S.); avirgolino@medicina.ulisboa.pt (A.V.); joao@campus.ul.pt (J.F.); pnogueira@medicina.ulisboa.pt (P.N.); andreiajsilvadacosta@gmail.com (A.C.); 2 Centro de Investigação e Estudos de Sociologia, ISCTE-Instituto Universitário de Lisboa (ISCTE-IUL), Avenida das Forças Armadas, 1649-026 Lisboa, Portugal; 3 Unbreakable Idea Research, 2550-426 Painho, Portugal; 4 Direção-Geral da Saúde, Alameda Dom Afonso Henriques, 1000-123 Lisboa, Portugal; carlotavieira@dgs.min-saude.pt (C.P.V.); mariajoaobg@dgs.min-saude.pt (M.J.G.); pedrograca@fcna.up.pt (P.G.); 5 Programa Nacional para a Promoção da Alimentação Saudável, Direção-Geral da Saúde, Alameda Dom Afonso Henriques, 1000-123 Lisboa, Portugal; 6 Faculdade de Ciências da Nutrição e Alimentação da Universidade do Porto, Rua do Campo Alegre 823, 4150-180 Porto, Portugal; 7 Laboratório de Biomatemática, Faculdade de Medicina, Universidade de Lisboa, Avenida Professor Egas Moniz, 1649-028 Lisboa, Portugal; 8 Escola Superior de Enfermagem de Lisboa, Avenida Professor Egas Moniz, 1649-028 Lisboa, Portugal

**Keywords:** food labelling, front-of-pack labelling, nutrition policy, health impact assessment, health literacy, health equity

## Abstract

This study aimed to describe the underlying process, used methods and major recommendations emerging from a comprehensive and prospective health impact assessment of the endorsement of a front-of-pack nutrition labelling (FOP-NL) system by the Portuguese health authorities. A mixed-methods approach was used to gather information on the impact of four FOP-NL schemes on consumers’ selection of food products according to the perception of their nutritional quality, combining a systematic literature review, focus groups (FG), in-depth individual interviews, and an open-label crossover randomized controlled study. The relevance of FOP-NL as a public health promotion policy has emerged as a consensual idea among either FGs’ participants (i.e., consumers and experts), or interviewed stakeholders. Although all of the evaluated FOP-NLs result better than no system on promoting the choice of the healthiest product, the effectiveness of easy-to-interpret FOP-NL among vulnerable groups raised concerns related to the need of integrating specific nutritional information to promote a better self-management of chronic diseases, and related to the level of literacy of consumers, which could impair the usage of FOP-NL. Educational campaigns addressing skills to use FOP-NL is recommended. Furthermore, a monitoring strategy should be considered to evaluate the long-term effectiveness of this policy in promoting healthier food choices, and in reducing diet-related non-communicable diseases burden.

## 1. Introduction

Noncommunicable diseases (NCDs) are increasing worldwide, accounting for more than 70% of all deaths, globally, and representing a leading cause of years of life lost across countries [1]. Almost 50% of premature deaths (or even more, if considering some types of cancer) are due to cardiovascular diseases and diabetes [2]. Recent data revealed that three of the five risk factors figuring as leading causes of disease burden are diet-related, namely high systolic blood pressure, high fasting plasma glucose, and high body-mass index [3]. These risk factors are linked to a shift in dietary patterns, mainly characterized by the increase of energy-dense, highly processed, and packaged foods, and related to the decrease of the intake of other healthy foods, such as whole grains, vegetables, and fruits [4]. These unhealthy diet behaviors constitute risk factors that take a determinant role in the increasing prevalence of overweight, and, consequently, in the causal chain of NCDs [5]. Though the consumption of ultra-processed foods are associated with worsen health status [6], guidelines for planning public health policies have recommended to intervene in the diet as a modifiable risk factor and promoting healthier dietary behaviors through creating healthy food environments [7].

Nutrition labelling has been pointed out as a pivotal strategy to modify the food environment [8,9]. This policy has the potential to enhance not only healthy food choices, throughout the provision of nutritional information to consumers, but also to encourage food reformulation operated by the food industry [10,11]. Since the first appearance of nutrition labelling in packaged food, the type of provided information and its presentation form have evolved [12]. The first forms of nutrition labelling presented the absolute content of a set of nutrients (e.g., calories, carbohydrates, fat, protein, or sodium), similar to the Nutrition Facts panel [12] or to the nutrition declaration in Europe [13]. More recently, the nutrition labelling evolved to provide interpretational aids (e.g., words, colors, or symbols) for helping consumers to quickly identify and consume healthier food options, namely front-of-pack nutrition labelling (FOP-NL) schemes [14]. The impact of this interpretive nutrition labelling systems has been studied: a meta-analysis found that FOP-NL systems can promote purchases of foods with lower sugar and sodium content in comparison to the condition where no label was available [15].

Recently, several FOP-NL systems have proliferated globally, being developed and/or promoted either by academic and public entities or by food industry operators [12]. Different types of FOP-NL emerged, namely endorsement logos, summary indicator systems, nutrient-specific warning labels, and nutrient-specific interpretive labels. The endorsement logos give an overall assessment of the absolute healthfulness of a product, with a positive evaluative judgment [14,16]. Additionally, summary indicator systems give an overall assessment of the relative healthfulness of a product, with both positive and negative evaluative judgment [14,16]. Moreover, nutrient-specific warning labels provide information about the surplus quantity of an individual nutrient, concerning a pre-established threshold, whilst nutrient-specific interpretive labels provide information about the quantity and relative percentage (i.e., low, medium, or high) of a set of individual nutrients [14,16]. Other systems have also combined, in a unique FOP-NL system, information which is provided by two of these types. Some FOP-NL examples are the Choices logo, an endorsement logo introduced in the Netherlands in 2006 and developed by the food and beverage industry [17]; the nutrient-specific interpretive system color-coded percentage of Guideline Daily Amounts (%GDA), mostly known as traffic light (TL) labelling, promoted by the Food Standards Agency, from the United Kingdom, in 2009 [18]; and the Nutri-Score (NS), a summary indicator scheme recently developed by the French public health agency, Santé Publique France, and endorsed first by the French government in 2017 [19], before being endorsed also by its homologous in other European countries, e.g., Belgium and Spain. Faraway, the Health Star Rating (HSR) is also used in Australia and New Zealand and is comprised of a summary indicator combined with a nutrient-specific interpretive component which also informs about the quantity of products’ nutrients (e.g., saturated fat, sugar, and sodium) [20]. To face the proliferation and use of different systems, in an unregulated way, several governments or food sector authorities in European countries have already endorsed the adoption of one single FOP-NL system for the food products that are commercialized in their territory [16].

In Portugal, several FOP-NL are currently used in the food packages which are commercialized by different food industry operators, but no single/unique FOP-NL has been yet adopted by the Portuguese government. The percentage of %GDA, a non-colored nutrient-specific system, was introduced in Europe and recommended by the food and beverage (F&B) industry representatives operating in Portugal, and then specifically adopted by a main Portuguese retailer operator. Following the adoption of the TL system by the United Kingdom, another main retailer operator in Portugal introduced this scheme in its own brand products. At the same time, other heretofore adopted systems (e.g., Nutri Pass) have been withdrawn from the market. Since the beginning of the year 2019, NS has been incorporated in the food packages of the brand products of another distribution operator. Consequently, multiple FOP-NL systems are now available in Portuguese food products, which may complicate their understanding and discourage their use by consumers [21], though a government-endorsed policy on interpretive nutrition labelling is still lacking.

This current situation in Portugal has important consequences. A report of the World Health Organization (WHO) stated that 40% of Portuguese consumers did not understand the nutritional information on food labels, a result that is even worse among those with low educational level (60%) [22]. Indeed, differences in effectiveness of FOP-NL policies on promoting healthy food behaviors among specific subgroups of the population have been documented, with a smaller effect being observed in lower socio-economic groups [23].

Though several health determinants can impact the usage of FOP-NL systems among different population subgroups, health inequities caused by the adoption of a given FOP-NL system should be taken into account to minimize their potential enhancement after this policy implementation. Thus, the Portuguese Directorate-General of Health considered as highly relevant to conduct an assessment of the potential health impacts regarding the adoption of a single FOP-NL and which FOP-NL would be more culturally adequate to promote healthy food choices, minimizing inequities. Taking this context into account, a health impact assessment (HIA) method was elected as the most useful approach to create an evidence-informed policy concerning the implementation of a proper approach on FOP-NL in Portugal. A HIA is a combination of procedures, methods and tools to assess the potential effect (or differences on the effect) of a policy on the population’s health (or subgroups of the population), in order to inform the decision-making process [24]. The policy proposal hereby assessed was the government-endorsement of a unique/single FOP-NL system. Therefore, the main goal of this paper is to describe the process of a prospective and comprehensive HIA, that aimed to evaluate different interpretive FOP-NL systems in terms of their potential to contribute for more informed food choices and, potentially, healthier food habits in the Portuguese population. The knowledge resulting from this HIA process informs policymakers in the decision of what FOP-NL system should be endorsed in Portugal. For this purpose, a brief description of the anticipated impact and a set of recommendations that should be considered to define and manage the policy implementation process are included in this paper.

## 2. Materials and Methods

### 2.1. Type of HIA and Management

This HIA was conducted between July 2018 and May 2019, with the scientific and technical guidance of the World Health Organization (WHO). According to the criteria defined by the Institute of Public Health in Ireland (IPH), a prospective and comprehensive assessment was carried out to inform the policy proposal development process with the best scientific available evidence [25].

A steering committee for the HIA was established before the beginning of the process. Two teams were composed, and the competencies of the members were defined. The HIA also entailed an advisory team which managed the HIA, was comprised by experts of several areas of expertise, such as nutrition, nursing, health care administration, and statistics. Finally, the project also included a core team, responsible for project execution, which was comprised of researchers with expertise in nutrition, psychology, and health sociology. The composition of both teams is detailed in the Appendixes, specifically in the Scoping tool.

Two workshops (one at the beginning and the other at the end of the project) were held with the purpose of engaging key stakeholders, representing the main affected groups and to elicit the ways the proposal would affect them.

### 2.2. HIA Process and Data Collection

**Figure 1:**
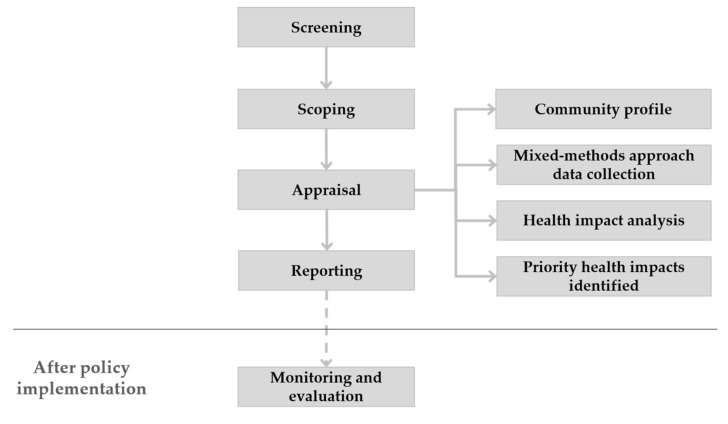
Stages of health impact assessment (HIA) process.

The methodology of the HIA process, as established by the IPH, involves a series of stages that are presented in the Figure 1. Furthermore, Table 1 describes how the different stages of this HIA exercise were conducted: (1) screening (decision whether to conduct the HIA or not); (2) scoping (establishment of a steering group and the definition of the terms of reference, as well as the work plan of HIA); (3) appraisal (gathering, consideration and prioritization of evidence of potential health impacts); (4) reporting (report of main findings and development of recommendations for the proposal implementation); and (5) monitoring and evaluation (establishment of HIA process monitoring as well as evaluation of impacts on health in the longer term) [25].

As the monitoring and evaluation stage is conducted after the policy implementation process, this paper only describes the stages which were operated before this moment. Notwithstanding, the process of medium- and long-term evaluation were already defined.

During the appraisal stage, a mixed-methods approach [26] was adopted to gather information and to estimate the potential health gains or losses of implementing the assessed proposal. The methodology was defined to accomplish a systematic literature review (SLR), two focus groups (FG) with experts and three FG with citizens, five in-depth individual face-to-face interviews with stakeholders, and a cross-sectional, open-label, crossover randomized controlled study with a random sample of the Portuguese population.

The SLR aimed to examine which types of interpretive FOP-NL schemes are effective in promoting healthier food choices and to assess these stated effects according to socioeconomic inequalities factors. For this second aim of the review, the PRISMA-Equity 2012 Extension (PRISMA-E) [27,28,29] was adopted, and the PROGRESS-Plus framework (i.e., an acronym which refers to place of residence, race/ethnicity/culture/language, occupation, gender/sex, religion, education, socioeconomic status, social capital, age, disability and sexual orientation) [30,31] was used to conduct an equity-focused analysis of the outcomes that were assessed in each of the selected studies. The inclusion criteria for the SLR were defined to gather the best available evidence regarding the effectiveness of interpretive FOP-NL systems on promoting healthier food choices. Longitudinal intervention studies, with an assessment of the outcomes of interest in pre- and post-intervention, were searched. Identified outcomes were related to healthier food choices, such as the impact on purchase intention, the consumers’ perception of healthiness of products, the nutritional quality of chosen products, the nutrient profile intake, the understanding of nutritional content and the effective overall caloric/nutrient intake. The complete description of methods of the SLR can be found in the article that has been published elsewhere [32].

Secondly, FG and individual in-depth interviews were performed aiming to characterize opinions about the effectiveness of an interpretative FOP-NL for improving consumers’ ability to obtain, interpret, and use the information of FOP systems. A psychologist and a nutritionist, who integrated the project team and had expertise in running FG, moderated and co-moderated, respectively, all the sessions. A third researcher registered field notes regarding nonverbal communication and group dynamics. For the FG with experts, professionals from crucial areas were identified and invited, namely, from nutritional sciences, health promotion, and health communication. For the FG with citizens, a set of heterogeneity criteria were considered to recruit people of different ages, educational levels, and social-cultural backgrounds. Several topics were defined relevant for the discussion, such as concerns about food choice, use of nutritional labels, interpretive FOP-NL systems (comparing different ones) and their potential impact as a food-choice determinant.

For the interviews, key stakeholders were identified to include representatives of the F&B industry and public authorities that regulate the food sector. The structure of the interviews focused the discussion of stakeholders’ perceptions about the determinants, obstacles, and facilitators for implementing an interpretative FOP-NL system, as well as their perspectives on its impact on food choice and food-related behaviors. The interviews were carried out by one researcher, with background in nutrition, in one-to-one appointments.

Finally, a quantitative survey was conducted with a sample of the Portuguese population (between 18 and 64 years old), following a random process for the generation of phone numbers aiming to compare the performance between FOP-NL in terms of salutogenic food choices. Participants were informed about the study and those who consented to participate were surveyed. The first component of the survey included a telephone interview using a Computer-Assisted Telephone Interview (CATI) system (469 participants were included in the analysis). Participants were then invited to answer to a web-based second component of the survey, where preferences regarding each assessed FOP-NL were assessed, using a questionnaire based on Julia and colleagues [20]. Participants were asked to choose the healthiest food product in each of five food choice scenarios (each one with a different condition: four FOP-NL systems plus a control condition with no-FOP-NL), composed of three product packages each. This exercise aimed to evaluate the impact of different pre-identified FOP-NL schemes on the selection of food products according to their perceived nutritional quality. Systems to be evaluated were established by a steering committee, as previously referred, following policy analysis, and considering systems already used in Portugal or other countries with similar socioeconomic contexts. The detailed methods of this survey were already previously described in the literature [33].

### 2.3. Ethical Considerations

The study was supported by the Portuguese Directorate-General of Health. The protocol was submitted to and approved by the Ethics Committee of the Centro Académico de Medicina de Lisboa (CAML) before all the participants’ enrollment and data collection processes. This study followed the Code of Ethics of the Declaration of Helsinki [34] for observational studies. For the focus groups, the in-depth individual interviews and the open-label crossover randomized controlled study, participants received detailed information about the goals, procedures, and average time of completion before enrollment. Participants were also informed that they could interrupt their participation at any moment and that their involvement would not require any effort besides answering the questions. Participants who agreed to participate signed a formed consent and received a duplicate of the document.

## 3. Results

This paper describes the process and the methods of a comprehensive and prospective HIA of endorsing of a FOP-NL system. The main conclusions of this HIA process, which used a mixed-methods approach for the appraisal stage, are herein summarized. Moreover, a set of recommendations that emerged from the evaluation process and that should be consider in the definition and implementation of a FOP-NL system are also shared as a relevant piece of information for policymakers.

### 3.1. Screening and Scoping

At the screening stage, undertaken between July and October 2018, the steering committee, comprised of both advisory and project teams, was established, and the potential health impact of the endorsement of a given FOP-NL system on health determinants, especially in population subgroups, was discussed by the steering committee. There was a consensus about the potential impact of the implementation of a FOP-NL system on social and economic conditions influencing health, namely education, childcare, and community interaction. Regarding structural issues affecting health, the potential impact of the proposal on public spaces, specifically grocery stores, supermarkets, or other selling-food places, was also consensual. Lastly, individual and family issues were also considered to be affected by the proposal, particularly the diet, the household income and the self-management of health and diet-related chronic diseases. The report of the screening stage and the rationale of the potential impact of a FOP-NL policy on health determinants can be consulted in Tables S1–S3.

Taking into account the evidence collected in the screening phase, it was decided to proceed with HIA. During the scoping stage, the steering committee met regularly to define the terms of reference, as well as the work plan of the HIA process. The scoping tool whit the report of this stage is available in Table S4.

#### 3.1.1. Policy Analysis

The policy analysis was conducted covering the Portuguese policy context on nutrition labelling. Policy on nutrition labelling in Portugal was framed by the European Union (EU) regulation No. 1169/2011, which defined as mandatory the use of the nutrition declaration (ND) on the back-of-package of commercialized foods in member states of the EU, i.e., nutrients in absolute quantities (e.g., saturated fat, sugar, salt, and trans-fat) by 100 g or 100 mL [13]. Interpretive FOP-NL were also allowed as an additional form of expressing nutritional information in an easy-to-use way for consumers to be voluntarily used (or not) by the industry [13].

The Integrated Strategy for Healthy Eating Promotion (EIPAS), in articulation with the National Program for Promotion of Healthy Eating (PNPAS), proposed the incentive to the use of FOP-NL schemes to ease food choices at the point-of-purchase [35]. In order to solve the multiple uses of FOP-NL by the F&B industry and retailers, and following the recommendation of EIPAS, in 2018 two political parties presented separated (and coincidentally) proposals for the use of the same FOP-NL scheme: the TL. Notwithstanding that both proposals have been rejected by the Parliament, at the time, a recommendation was done to the Portuguese government to study the endorsement of a single FOP-NL system [36].

So far, appeals have been made by academics and other representatives of public health entities regarding the need to regulate this area and to endorse the use of a unique/single FOP-NL by the F&B industry operating in Portugal [37]. However, these positions have proposed the endorsement of a given FOP-NL system (the NS, in this case) whose effects on promoting healthier food choices were not studied for Portuguese consumers yet [38]. Consequently, evidence to sustain the decision-making process is still lacking.

For these reasons, the steering committee decided to evaluate the effect of and the perspectives about four FOP-NL, among the Portuguese population, namely TL, %GDA, NS and HSR according to the following rationales: TL, %GDA, and nowadays NS, are already used in the Portuguese population by three of the major food retail operators in Portugal; HSR (endorsed by Australian and New Zealand authorities) is not known by Portuguese population and combines an overall nutrition summary with a set of nutrients-specific assessments, both complementing limitations of each other.

#### 3.1.2. Stakeholders Engagement in the HIA Process

Stakeholders and representatives of those considered to be the most affected groups of the population (by the potential implementation of this public health measure) were engaged in the process through two meetings. The first took place before the beginning of the appraisal stage of HIA, on 28 January 2019, assembling nine institutions of the invited ten. This workshop was comprised of representatives of consumers, the F&B industry, health sector professionals, food sector regulators, academy, and policymakers. Stakeholders were introduced to the proposal, informed of the aims and the work plan of this HIA, and actively involved in the process. Subsequently, to the execution of the HIA, the stakeholders previously invited plus one were asked again and nine met on 25 October 2019, to be presented with and to discuss the obtained results. Stakeholders who participated in the workshops valued their involvement and highlighted the pertinence of conducting a HIA to inform policymakers on this proposal. Moreover, the methodology of data-collection was broadly praised, and the relevance of the achieved results was considered crucial for evidence-based policymaking. Stakeholders proposed sharing the results with the European Commission to inform the potential decision on the endorsement of a FOP-NL to be adopted by the F&B industry and retailers operating in the European Union member states territory.

### 3.2. Appraisal

#### 3.2.1. Community Profile

The appraisal stage started with the description of the baseline health status of the Portuguese population, particularly specific subgroups that could be differently affected by the proposal. In Portugal, about 30% of children aged between 6 and 8 years are overweight [39], a value that is even more alarming among adults, with about 67% being overweight. The prevalence of obesity, and other diet-related NCDs, such as diabetes and hypertension, is higher among those with lower educational or income levels [39,40]. Moreover, the daily consumption of fruit and vegetables is insufficient in more than half of the Portuguese population and more pronounced in children (72.0%) and adolescents (78.0%). In addition, the proportions of the population with a high intake of saturated fat, sugar, or sodium above the recommendations are in the same order 53.0%, 24.0%, and 89%, respectively. Furthermore, the subgroups with the lower education level, which present higher prevalence of some health conditions or diseases, are also those with higher proportions of individuals who do not understand the nutritional information [22].

#### 3.2.2. Impact Assessment

For the appraisal stage, the mixed-methods approach for data collection was comprised of an SLR, focus groups, individual in-depth interviews, and a survey. Regarding the SLR, nine studies were reviewed, and their qualitative analysis studies revealed that, in general, interpretative FOP-NL systems were found to have a beneficial impact on healthier food choices when compared to a no-label condition. More detailed results of systematic literature review were published elsewhere [32].

Overall, 31 participants were involved in five FG: two FG on 3 and 4 October 2018, with 11 experts (*n* = 6 and 5; 20 were invited), and three FG with 20 citizens (*n* = 8, 7 and 5; 26 were invited), on 19 September, 3 and 4 October 2018. Five in-depth individual face-to-face interviews took place between 26 December 2018 and 19 February 2019.

Finally, the survey data collection was performed between 12 February and 31 March of 2019. A total of 469 participants answered the first component of the survey (CATI-based survey) and 357 participants participated in the second component (self-administrated web-based survey). All the four FOP-NL systems that were assessed in this second component of the survey, i.e., %GDA, TL, NS, and HSR, performed better than a no-FOP-NL condition, aiding the selection of healthier food products. Results from this survey were also published elsewhere [33]. A general summary of the results of the SLR, qualitative data (i.e., FG and interviews), and the survey are available in Table 2.

**Figure 2:**
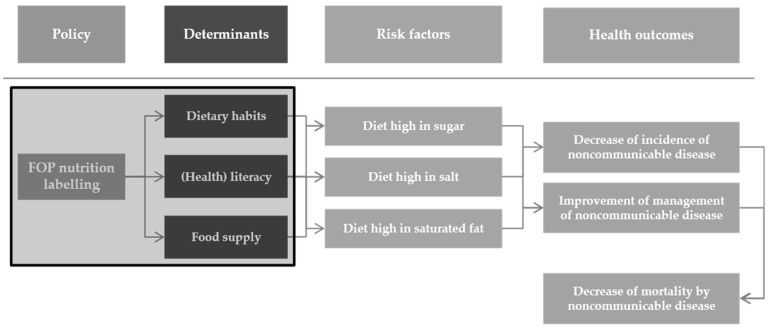
Causal pathway of front-of-pack (FOP) potential health impact.

After the analysis of qualitative and quantitative data, which was gathered through the already described mixed-method approach, the causal pathway of the impact of the implementation of the endorsement of a FOP-NL system was delineated, as presented in Figure 2. The box highlights the determinants which were evaluated in this HIA.

### 3.3. Recommendations

A set of recommendations were delineated to be potentially considered by policymakers during the implementation of the proposal, namely the endorsement of a single/unique FOP-NL system to be presented on food products, commercialized in Portugal. These recommendations are presented in Table 3.

### 3.4. Monitoring and Evaluation of the HIA Process

The evaluation of the policy implementation should also be strategically defined, stipulating the monitoring of the effect of the proposal before its implementation. In this case, the outcomes of the implementation of a FOP-NL system should be monitored and evaluated in a short- and medium-term, but longer-term outcomes can also be considered even though they are affected by many other factors [53,54]. A set of assessment methods and indicators to assess after the implementation of the proposal was proposed:

- Monitoring and characterization of the magnitude of the adoption of the endorsed FOP-NL system, and its presentation on food products package, by F&B industry;
- The effectiveness of FOP-NL impact on purchasing decisions and overall diets in real-life research scenarios should be evaluated (e.g., interventions comparing nutritional quality of real purchased food products—for example, in hyper/supermarkets—with or without the FOP-NL presented in food packages);
- Monitoring the potential changes on food composition of products after starting to use the endorsed FOP-NL, though the potential of FOP-NL to originate a food composition reformulation (possibly to improve the classification attributed by a given FOP-NL system algorithm);
- Conducting a retrospective HIA to evaluate the impact of this proposal after five years of implementation should also be considered, focusing on food choices in point-of-purchase contexts and, ultimately, in food habits (e.g., analyzing the Portuguese food balance or household budget survey);
- The incidence or prevalence of diet-related diseases, as well as (premature) mortality by these causes, are more distal expected outcomes of this policy which should be considered within a continuous and long-term monitoring assessment system of the impact of this health policy initiative. Inequalities on these indicators among subgroups of the population should also be observed and analyzed.

## 4. Discussion

A prospective and comprehensive HIA was conducted, according to the procedures described by the IPH, to inform Portuguese policy-makers about the decision of which FOP-NL system to endorse in Portugal. Data collected during this HIA appraisal stage followed a mixed-methods approach, which included a SLR, FGs, in-depth individual face-to-face interviews and a survey. Main findings of the SLR suggested that FOP-NL systems lead to better results in several outcomes related to a better understanding of nutrition labelling or better nutritional quality of food choices, even though no particular system emerged as most effective than others. This proposal of endorsing a unique FOP-NL system was consensually perceived as a relevant public health promotion policy among citizens, experts, and stakeholders. Moreover, the evaluation of the impact of four FOP-NL systems within a sample of the Portuguese population revealed that all systems allowed higher rates of selection of healthy food choices, in comparison with the no-nutritional label control condition. The TL was the preferred FOP-NL system among the surveyed participants, regarding several positive characteristics, e.g., useful about the provided information, trustworthiness, reliable, and being easy to understand and quick to process.

To the best of our knowledge, this is the first HIA evaluating a policy proposal concerning the government-endorsement of a FOP-NL system. Notwithstanding, the HIA method was already used to evaluate similar proposals, such as the impact of mandatory restaurant menu nutrition labelling on population weight gain, which found a benefic impact of this policy on the obesity epidemic [58]. Following the recommendation of the European Commission for considering the evidence as a core value for informing policy-making [59], the undertaken HIA constituted a fundamental process in the development of evidence-based policy on nutrition labelling and evidence-based recommendation for its potential implementation. Indeed, the HIA methodology comprises relevant characteristics for the evaluation of a health policy proposal, especially when it can potentially affect differently and generate inequities among higher-risk population subgroups, as labelling policy is proposed to do [16]. The decision to undertake a comprehensive HIA was justified by the need to collect a broad range of scientific evidence [25], not only regarding the effectiveness of different FOP-NL systems on the interest outcomes but also regarding the opinions and preferences of the Portuguese population on FOP-NL. The mixed-methods approach, which was followed in the appraisal stage of this comprehensive HIA, allowed the integration of qualitative (i.e., collected on FG, individual in-depth interviews) and quantitative (i.e., a telephone and web-based survey) methods to collect evidence to inform and to sustain a decision of policy-makers on which FOP-NL system to endorse [25,26].

Conducting an HIA, in this case to evaluate the impact of the endorsement of a FOP-NL system by the Portuguese government, can promote health gains, mitigate negative health impacts and reduce/prevent health inequalities [25,60]. The performed SLR aimed to review the best evidence about the effectiveness of FOP-NL systems and to inform the decision of what systems should be considered to endorse. Despite good performance of several FOP-NLs in achieving healthy food choices, contradictory results were found regarding which was the most effective one. A recent narrative review also suggested that FOP-NL systems can help consumers to differentiate what foods are more or less healthy [51]. However, the author identified warning labels as the most successful system in the increasing of the intent to purchase healthier foods [51]. The need to focus this HIA on health equity through the observation of the differential effect of FOP-NL on higher risk population subgroups was also considered in the decision of using selected methods, namely the equity-focused SLR [27,28,29] following the PROGRESS-Plus framework [30,31]. Nonetheless, facing the small number of reviewed studies which evaluated the effect of FOP-NLs according to equity determinants, and the heterogeneity of their data collection, more evidence is still necessary to determine the role of several systems in decreasing inequities. The same conclusion was already advanced in a meta-analysis which noticed the need for further research to understand how purchasing and consumption are affected by specific FOP-NL systems among the most vulnerable subgroups [15].

Despite a good acceptance of a FOP-NL endorsement proposal, a broader knowledge about the effect of different FOP-NLs in people belonging to lower socio-economic subgroups of population was also advocated, by the participants in FGs and interviews, to guide the decision of what system to endorse. This concern is not exclusive of this study: the need to address and to consider these differences of effect were already highlighted during a process of nutrition labelling-related policies implementation in Latin American countries [61]. Citizens, experts, and stakeholders consensually called attention for the importance of a health/nutritional education program to promote the usage of the implemented FOP-NL and to prevent health inequities, as a result of this policy. The inclusion of an educational campaign in the implementation strategy would enhance the understanding and use of the endorsed FOP-NL, and is common throughout previous implemented systems [53]. In line with European legislation, none of FGs participants or interviewed stakeholders defended the mandatory adoption of the endorsed FOP-NL system. However, a strategy to avoid a selective adoption was already proposed in Australia and New Zealand during the HSR implementation process. In those countries, aiming the widespread adoption of using this FOP-NL system, efforts are being made to achieve the required display of HSR in 70% of target products within five years (until the end of 2023); otherwise, authorities will consider mandatory adoption [62].

Despite the endorsement of FOP-NL systems by several European countries, based on evidence for their population (e.g., TL on United Kingdom [18] or NS on France [19]), evidence regarding the easiness to interpret FOP-NL systems, the effectiveness of FOP-NL systems on promoting healthier food choices, and regarding which system performs better, was still lacking for the Portuguese setting. The results of the survey supported that FOP-NL systems can help Portuguese consumers to make healthy food choices, with a (non-significant) tendency for TL to perform better than the other evaluated systems. A subsequent study found similar results regarding the ability of different FOP-NLs (i.e., TL and NS) to improve nutritional quality of food choices among Portuguese consumers in comparison with no label condition. In that study, NS was the most effective system in improving participants’ ranking ability [63]. Notwithstanding, and before the conduction of these two studies about the effectiveness of FOP-NL for the Portuguese population, appeals [37,38] were made to proceed with the endorsement, in Portugal, of the NS (i.e., without previous evidence about its effect on promoting healthier choices in the Portuguese population). Similar to the no-evidence discussions previously observed before or during the implementation of a FOP-NL system [64], this discussion can be a driver of positions polarization and can act as a barrier to define and implement an evidence-based FOP-NL policy. Thus, this HIA is a contribution to overcome the lacking evidence for informing the development of an evidence-based policy on FOP-NL taking into account and adjusting the proposal to the Portuguese population.

Several strengths are attributed to the HIA method. The involvement of stakeholders, a key strategy of a HIA process, allowed to involve citizens, experts and representatives of the F&B industry, food retailers and regulatory agencies, as all these social actors have contextual knowledge that could lead to the provision of relevant insights about the potential impact of the proposed policy. Furthermore, they could also contribute to the identification of population subgroups that may be differently affected by the proposal, thus anticipating the need to consider particular details during its implementation, in such a way that can lead to increased health gains and reduced social inequalities [65]. The Portuguese population profile was described to set a baseline which allows further comparisons, after the proposal implementation. The described characteristics, in terms of health status and food and nutrients intake, are very similar to other European countries. Regarding the proportion of overweight (including obesity), Portugal stands in the worst situation among the European countries that comprises The Organization for Economic Co-operation and Development (OECD) [66]. Portugal has also one of the highest proportions of individuals living with two or more chronic conditions in the OECD, only below the Finnish population [66]. Furthermore, Portugal compares with most of the other European countries, where a poorer health status is more common among those with lower educational or income levels [66]. The same for food behavior: the insufficient intake of fruit and vegetables is also observed for most of European citizens, including Portuguese [67]. Summing up, unhealthy dietary habits are not exclusive for Portugal, which means that concerted policy development at regional level are crucial to promote healthy food environments and achieve a systemic dietary behavior improvement in Europe [7].

This study faced some limitations that are relevant to address. The sample size obtained with the survey impairs the capacity to generalize to the Portuguese population our findings considering the effectiveness of four assessed FOP-NL systems on promoting healthier food choices. Notwithstanding, the characteristics of the sample surveyed were very similar to the Portuguese population concerning sex and education level [68]. Moreover, the main goal of the survey was to compare the performance between FOP-NL in terms of salutogenic food choices and this was done with a heterogeneous sample, allowing to address the important issue of the social equity of a public health measure.

Regarding the proposed monitoring indicators, data on diet-related morbidity should be carefully analyzed due to the difficulty of associate its potential changes specifically to the effect of a single proposal, i.e., the endorsement of a FOP-NL system, and this is an identified limitation of this HIA. However, the expected potential of this proposal for changing dietary behaviors (e.g., high intake of sugar, fat, or salt) justifies the relevance of monitoring medium/long-term epidemiology of NCD’s in the country and associated premature mortality.

## 5. Conclusions

This HIA showed that the relevance and beneficence of government endorsement of a unique/single FOP-NL system is rather consensual among citizens, experts, and stakeholders. All the evaluated FOP-NL systems performed better than the no-nutritional label condition and can potentially act as an inductor of healthier food choices (i.e., a health nudge). However, no statistically significant differences were observed between the performances of each system, despite a higher number of correct choices was reached when participants were asked for selecting the healthiest food package from a set of three alternatives with TL being presented. The implementation of an endorsed system should be accompanied by an effective educational campaign (promoting adequate health literacy to use properly the adopted FOP-NL) and monitoring the potential changes in food composition of products after starting to use the endorsed FOP-NL.

The health impact of the food policy measure which was assessed in the scope of this HIA may become only clear after several years after its implementation. Therefore, a monitoring system (allowing the assessment of the effectiveness of this health policy, also from a health/nutritional equity perspective) should also be considered. Further research should also contemplate long-term studies to assess how the implementation of a FOP-NL would impact major health outcomes, namely morbidity and mortality by diet-related noncommunicable diseases.

## Figures and Tables

**Table 1:** Stages of HIA process and respective activities.

HIA Stage	Activities
**Screening** July–October 2018	The proposal was defined, namely, the endorsement of a unique/single front-of-pack nutrition labelling (FOP-NL) system by the Portuguese government; Potential impacts of the proposal on health determinants were described; Vulnerable groups of the population most likely to be affected by the proposal were identified.
**Scoping** October–November 2018	The work plan for the HIA was established; A policy analysis aiming to frame the policy context of nutrition labelling was conducted, focusing also FOP-NL systems which were used, at a national and international level; FOP-NL systems whose impact would be evaluated were defined in the scope of this HIA.
**Appraisal** November 2018–April 2019	The Portuguese population health profile was identified; Information on potential impacts (health gains or losses) of the implementation of the proposal was gathered; A mixed-methods approach was followed for data collection, including a systematic literature review, focus groups with consumers and experts, in-depth individual face-to-face interviews with stakeholders and a survey with a random sample of the Portuguese population.
**Reporting** April–May 2019	Main findings were reported to decision-makers, stakeholders and other affected groups; A set of recommendations was detailed to modify the health impacts of the proposal implementation (maximizing health gains and minimizing health losses).
**Monitoring and evaluation** July 2018–5 years after proposal implementation	The HIA process was monitored during the period of prosecution; The evaluation of the implementation of the proposal was also projected.

**Table 2:** Main findings on potential impacts of the proposal, gathered through a mixed-method approach.

Data Collection Method	Main Findings
**Systematic literature review**	Benefits of interpretive FOP-NL systems were observed in the following outcomes: perception of products’ healthiness [41,42,43,44], understanding of nutritional content [41,45], purchase intention [41,42,46], nutritional quality of selected products [45,47,48,49], and nutrient content [45,47,48]; No particular system stood out as clearly the most effective, as each system is more helpful in some health-related dimensions but not in others; The most commonly assessed factors of social inequalities, according to the PROGRESS-Plus framework, were sex, age, education level and socioeconomic status; Nonetheless, more evidence is still necessary to determine the role of FOP-NL in decreasing inequalities among the most vulnerable subgroups through the promotion of healthy food choices.
**Focus groups and in-depth individual face-to-face interviews**	A consensus was found among citizens, experts, and stakeholders about the relevance of the proposal as a public health promotion policy, though the need for a program of health/nutritional education associated with its implementation; Experts and stakeholders interviewed agreed about the need for promoting widespread awareness and the voluntary rather mandatory adoption by the food and beverage industry, as well as about the need for evaluation of this policy impact; Experts and consumers expressed concerns about the algorithm for FOP-NL classifications, while stakeholders its widespread communication to increase the perception of transparency and avoid these concerns; Stakeholders referred to as being concerned with the potential increment of costs and logistics related to the implementation of FOP-NL, so they mentioned that their involvement in the entire process would be crucial; Guideline Daily Amounts (%GDA) was consensual as comprising information about nutrient amounts and relevant for self-management of health conditions, although more time could be needed to process it; population subgroups less educated or with lower levels of functional health literacy would benefit less; Traffic light’s (TL) colored nutrient amounts were considered familiar to the Portuguese population and considered ease and adequate to be interpreted and used by subgroups with lower levels of literacy, as well as those who need to self-manage health conditions; TL does not allow a direct overall summary for comparisons between products; Nutri-Score (NS) was perceived as an evaluative summary score which does not require a high level of health literacy to interpret, potentially enhancing equity on vulnerable groups; concerns about the characteristics of the algorithm (adequate/inadequate dichotomy), as well as about the lack of information regarding nutrient amounts for consumers’ self-management of health or diseases; Health Star Rating (HSR) was understood as a combination of summary score and nutrient-specific types of information, which can decrease time to process it; nevertheless, it was perceived as useful for groups with low literacy skills or for groups for which this information helps to manage health conditions, the use of stars was not appreciated by several experts and consumers.
**Survey**	469 individuals were interviewed, while 357 completed also the web-based component of the survey; %GDA was recognized by 83.5% of the Portuguese population surveyed, followed by TL (82.6%), NS (16.2%), and last HSR (14.3%); TL was preferred, rather than other systems, in eight of the 12 categories of the questionnaire of Julia and colleagues [50], all describing positive characteristics of a FOP-NL. Otherwise, NS had the highest proportion of responses in three of the 12 categories, although these items reveal negative feelings; TL reached the highest number of correct answers of individuals choosing the healthiest option informed by this system (72.3%), followed by the HSR (70.9%), the %GDA (70.0%), and NS (62.2%). The control condition with no FOP-NL being presented reached 34.2% of correct answers.

**Table 3:** Recommendations (and supporting evidence) for the process of implementation of the proposal.

Recommendations	Outcomes	Reference
A single/unique FOP-NL system should be endorsed by the Portuguese Government as a relevant and evidence-based public health policy. The decision of which FOP-NL system to endorse should consider the evidence and conclusions resulting from the HIA performed (namely, about the pros and cons of each FOP-NL system), as well as the FOP-NL systems adopted by other countries with expression at food trade level with Portugal	It is expected that the endorsement of a single/unique FOP-NL system could enhance the usage of the implemented one, potentially impact promoting healthy food choices and diminish a potential confusion effect of comparisons across label formats.	[21]
To prevent health inequities, the decision about which FOP-NL system to endorse should consider potential differences of effect in promoting a better understanding of food products’ nutritional quality and/or better food choices among population subgroups, namely those with specific nutritional needs	According to the evidence, including (preferably interpreted) information about certain substances (e.g., salt/sodium or sugar) which are related to the risk of the most prevalent non-communicable diseases (e.g., hypertension or diabetes) can lead to lower consumption of foods containing these nutrients, potentially contributing for the improving of chronic disease self-management.	[51,52]
In line with European legislation, the adoption by the food industry may be voluntary. However, rules on the adoption should be redefined to avoid the selective implementation of the endorsed system.	Although the European legal context, specific rules or a medium-term transition for mandatory adoption would help to overcome the selective adherence to the endorsed FOP-NL system (e.g., an operator interested in the implementation of the FOP-NL system in a food product must implement it in all own commercialized products, rather than voluntarily implement it only in the healthiest products).	[53,54]
The concerns expressed with the classification algorithm of some FOP-NL systems (e.g., NS) should be analyzed and eventual improvements should be considered (e.g., assessing its validity for classifying a basket of food products commercialized in Portugal to test its suitability to Portuguese food products)	Despite the discriminating performance of summary systems, namely Nutri-Score, was already tested within 921 Portuguese products, a broad study of specific products would allow to identify and to correct identified discrepancies [55] on classifications of less healthy products.	[53,56]
A strategy for the communication of the endorsed FOP-NL system (i.e., addressing and promoting knowledge and skills for its correct use) should be defined and all interested or affected stakeholders should be involved and engaged. Consumers or communities at higher risk of inequalities should be identified and should receive differentiated education according to their needs	It is expected that the wide promotion of this initiative, in an open and transparent process, can increase the knowledge about the endorsed FOP-NL and confidence among consumers and increase its usage. Though the potential beneficial effect of FOP-NL among most vulnerable groups of population promoting healthy dietary habits and the higher susceptibility of certain groups to use it (e.g., women and people with higher levels of nutrition knowledge), a communication strategy-oriented for specific characteristics of different groups can decrease potential differences of impact across different categories of consumers.	[53,57]

## Data Availability

Not applicable.

## Supplementary Materials

The following are available online at https://www.mdpi.com/1660-4601/18/4/1422/s1: Table S1: Section one: background and context; Table S2: Section two: potential impacts on health determinants; Table S3: Section three: screening outcome and Table S4: Scoping tool.
