# Supplementary material for: Looking Ahead: Health Impact Assessment of Front-of-Pack Nutrition Labelling Schema as a Public Health Measure
Source: Int J Environ Res Public Health. 2021 Feb 3;18(4):1422. doi: 10.3390/ijerph18041422 (PMC7913662; doi:10.3390/ijerph18041422)
Supplement: Supplementary file 1 [file ijerph-18-01422-s001.pdf]

International Journal of
Environmental Research and Public Health

MDPI

Supplementary Materials S1

Screening tool - Improving Nutrition Labelling in Portugal

Introduction

Health promotion actions must contribute, among other endeavours, to the creation of social and physical environments that support people to make healthy choices [1, 2]. A potential positive health impact can be achieved with intersectoral interventions, such as, for example, intertwining the healthcare sector with the food industry and companies. Nutrition information provided in labels about the composition of foods is a mandatory and important tool to support consumers' informed decisions [1] enhancing health gains.

Health Impact Assessment (HIA) is '*a combination of procedures, methods and tools by which a policy, program or project may be judged as to its potential effects on the health of a population, and the distribution of those effects within the population.*' The '*general objective of such assessments is to improve knowledge about the potential impact of a policy or programme, inform decision makers and affected people, and facilitate adjustment of the proposed policy in order to mitigate the negative and maximize the positive impacts*' [3].

Screening

Screening helps to determine whether an HIA is appropriate or necessary. Overall, it can contribute to the identification of:

- potential health impacts of a policy, programme or project;
- potential impacts on vulnerable sections of the population;
- the need for a more detailed assessment;
- the best way to effectively address health and equity issues using the HIA [4].

The screening tool developed by the Institute of Public Health in Ireland (IPH) [4] was used in the first phase of this HIA.

Table S1. Section one: background and context

**Title of proposal being screened**	A contribution for the impact assessment of a new initiative of nutrition labelling in Portugal (Nutr-HIA)
**Date screening conducted**	13th to 15th November 2017, 6th July 2018 & 10th October.
**Persons involved in the screening process**	• Pedro Graça Maria João Gregório (National Programme for the Promotion of Healthy Eating, Directorate-General of Health);<br>• Catarina Sena, Andreia Costa, Paulo Nogueira, Carlota Pacheco Vieira, Ana Paula Soares (Directorate-General of Health: DGS);<br>• Violeta Alarcão, Rodrigo Santos, João Fernandes, Ana Virgolino, Osvaldo Santos (Environmental Health Institute, Lisbon School of Medicine: ISAMB-FMUL).
**What stage of development is the proposal at?**	The proposal is being designed.
**Briefly outline the importance of the proposal from:**<br>An economic/ business perspective<br>A political perspective<br>A community perspective	1- Implementation of front-of-pack nutrition labelling (FOP-NL) has the potential to reduce the economic burden associated with chronic health conditions (which usually leads to considerable medical expenses, loss of work time, social and emotional problems, and even premature death, among other).<br>2- Adequate FOP-NL information may promote competitive advantage to food companies by attracting well-informed consumers.<br>3- The adoption of nutrition labelling on FOP-NL of prepacked foods may promote consumers' adequate/healthy choices at the moment of buying products (health decisions at the right moment).<br>4- FOP-NL has the potential to promote healthy habits formation at community level, acting as cues for action at strategic places/momentum.
**What resources are available to conduct a HIA?** (Consider both human and financial)	The Directorate-General of Health, the promoter of this project, has provided a budget to support all expenses associated with this HIA.<br>A team was formed with 10 elements with different profiles and expertise related to the theme, namely researchers, nutritionists, statisticians, health psychologists, sociologist, health managers, dedicated to the project for the next six

	months. Some researchers were subcontracted to the Faculty of Medicine, University of Lisbon, for having experience in qualitative and quantitative methodologies used in HIA related projects and in health promotion and health literacy.
**Are decision makers likely to be open to recommendations to amend the proposal?**	In Portugal, representatives of the Ministry of Health working in this area have recently reported that they intend to discuss the interpretative labelling system that should be used by all operators who have a genuine motivation to better inform consumers. On the other hand, the National Strategy for the Promotion of Healthy Eating (EIPAS), composed of seven ministries, has decided to contribute to the discussion on the evaluation, identification and eventual implementation of an additional nutritional labelling model (with interpretative coding on food labels) that facilitates an informed choice, supported by available scientific evidence.<br>This exercise will serve for the Directorate-General of Health to present to the Government the results achieved, their advantages and disadvantages.

Table S2. Section two: potential impacts on health determinants

**Social and economic conditions that influence health**		
**Likelihood that the proposal will impact on this health determinant**		**Groups most likely to be affected by the proposal**
Education	**L**	Children and young people, benefiting from integration of this new FOP-NL topics in their school curriculum.<br>Older people, benefiting from easy and useful information at the moment of prepacked food and drink purchase.<br>Individuals with low health literacy and/or low educational level (reducing health literacy gap).
Employment	**U**	
Childcare	**L**	Kindergartens and nursery schools' food purchase might be influenced with the higher awareness of the available nutritional options for the children due to the use of FOP-NL.
Crime and fear of crime	**U**	
Community interaction	**L**	Interpretive FOP-NL has the potential to spread new purchase habits among community elements (social imitation processes) from children to working age and older people – transgenerational perspective; from producers/industry to health professionals, health opinion-makers, individual or collective buyers.
Access to fresh food	**U**	
Access to sports and other opportunities for physical activity	**U**	
Access to cultural and other recreational activities	**U**	
Access to healthcare services	**U**	
Access to social welfare services	**U**	
Access to other community services	**U**	

Access to public transport	**U**	
Other social or economic conditions (list)	**-**	
**Structural issues that influence health**		
**Likelihood that the proposal will impact on this health determinant**		**Groups most likely to be affected by the proposal**
Housing	**U**	
Public buildings	**U**	
Commercial buildings	**U**	
Green space (including parks)	**U**	
Other public spaces	**L**	Public spaces selling food (e.g., supermarkets, grocery stores).
Road safety	**U**	
Transport infrastructure	**U**	
Communications infrastructure (internet/telephone)	**U**	
Energy sources	**U**	
Waste management infrastructure	**U**	
Water quality	**U**	
Air quality (indoor and outdoor)	**U**	
Soil quality	**U**	
Noise	**U**	
Light	**U**	
Other structural issues (list)	**-**	
**Individual and family issues that influence health**		
**Likelihood that the proposal will impact on this health determinant**		**Groups most likely to be affected by the proposal**
Diet	**L**	All food consumers.<br>Consumers with unbalanced/unhealthy eating habits.

		Citizens having dependent children.<br>Individuals with pre-obesity/obesity or with other chronic diseases requiring control/reduction of weight.
Physical activity	**U**	
Substance use (legal and illegal)	**U**	
Sexual activity	**U**	
Household income	**L**	Economic asymmetries can condition food choices of households. FOP-NL might set a similar basis for different economic strata.
Family cohesion	**U**	
Other individual and family issues (list)	**-**	Increase of autonomy and self-confidence for self-management of health and chronic diseases nutrition related.

Likely (L) = It is likely that the proposal will impact on this health determinant; **Unlikely (U)** = It is unlikely that the proposal will impact on this health determinant; **Not Known (NK)** = There is insufficient information in the proposal to assess whether or not it will impact on this health determinant.

Table S3. Section three: screening outcome

Overall, health impacts are unlikely or relatively minor and easy to address.	Where appropriate, make recommendations to decision makers on how such impacts may be addressed. Do not proceed with HIA.	
Overall, health impacts are likely or unknown.	Taking into account issues raised in section one, proceed with HIA.	Yes

Scoping tool - Improving Nutrition Labelling in Portugal

The scoping tool developed by the Institute of Public Health in Ireland (IPH) [4] was used in the second phase of the HIA of the current proposal. The blueprint and management of the HIA were defined in this stage, establishing the work plan for the rest of assessment.

Available online at http://www.publichealth.ie/hia.

Table S4. Scoping tool

**Title of the proposal on which the HIA is being conducted**	A contribution for the impact assessment of a new initiative of nutrition labelling in Portugal (Nutr-HIA)
**Aim of the HIA**	This HIA evaluates the consumers' perception and understanding of different front-of-pack nutrition labelling (FOP-NL) systems and analyse socioeconomic differences in the consumers' ability to understand it. This information aims to inform the decision-making process on the selection of a FOP-NL nutrition label to be recommended by the government as a complementary form of nutrition labelling.
**Values underpinning the HIA**	• Equity<br>• Evidence-informed<br>• Transparency<br>• Neutrality
**Objectives of the HIA**<br>(Consider core values)	1) To identify, through a literature review, recommendations for interpretive nutrition labelling, considering the cultural, socioeconomic diversity and the legal Portuguese-specific context.<br>2) To characterize, through focus groups and interviews, experts, stakeholders and citizens' opinions about the effectiveness of an interpretative front-of-package nutrition labelling for improving consumers ability to obtain, interpret, and use information of FOP-NL interpretative nutrition labelling.<br>3) To provide updated recommendations, on basis of the literature review and stakeholders (including health professionals, consumer associations, industry) & citizens' perspectives, for future legislation on nutrition labelling.

**Boundaries of the HIA**<br>(e.g., geographical, population)	Portuguese national policy<br>Multisectoral groups<br>Diverse health literacy level of the population<br>(in)Equity of access to food products
**Time scale for the HIA**	Final recommendations and report to be presented to World Health Organization by March 2019
**Non-negotiable aspects of the proposal**	Not applicable
**Steering group membership**<br>Suggest maximum of 12 members<br>Include decision makers of the policy, programme or project on the group	**Steering group**<br>Chair: Pedro Graça, Nutritionist, PhD (DGS) & Andreia Costa, Nursing, PhD (DGS)<br>Advisory team:<br>• Carlota Pacheco Vieira, Health Administration, MSc (DGS)<br>• Catarina Sena, Health Administration, MSc (DGS)<br>• Maria João Gregório, Nutritionist, PhD (DGS)<br>• Paulo Nogueira, Statistician, PhD (DGS)<br><br>Project team:<br>• Ana Virgolino, Psychologist, MSc (ISAMB-FMUL)<br>• João Fernandes, Nutritionist, MSc (ISAMB-FMUL)<br>• Osvaldo Santos, Psychologist, MSc (ISAMB-FMUL)<br>• Rodrigo Santos, Nutritionist, MSc (ISAMB-FMUL)<br>• Violeta Alarcão, Sociologist, PhD (ISAMB-FMUL)
**Main stakeholders**<br>Who is likely to be affected?<br>Are key stakeholders represented on the steering group?	• Consumers - represented<br>• Food and drinks industry / food and agriculture sector - represented<br>• Health professionals – represented<br>• Policy makers - represented
**Key informants for the HIA**<br>Who can provide useful information on how the proposal is likely to impact on health?	• Consumers (women & men, young & older adults, high & low education, high & low socioeconomic groups)<br>• Consumer associations<br>• Food and drinks industry / food and agriculture sector<br>• Health professionals<br>• Policy makers

**Who will be responsible for gathering evidence in the following areas?**<br>Literature review<br>Community profile<br>Stakeholder workshops<br>Proposal and policy analysis	Literature review: Project team<br>Community profile: Advisory group<br>Stakeholder workshops: Steering group<br>Proposal and policy analysis: Project team & Advisory group
**Who will be responsible for appraising the evidence and forming recommendations?**	Project team
**How will the results of the HIA be presented and disseminated?**	Results were presented to the World Health Organization and policy makers, through a technical report, with key-points' executive summary. Moreover, results were also disseminated throughout scientific community.
**What measures will be put in place to facilitate evaluation of the HIA?**	Measures used within current HIA:<br>• Impact/outcome evaluation to be decided<br>• Structured individual interviews and focus groups<br>• Survey with food-choice tasks, with or without front-of-package interpretative information, crossed by health indicators<br>Measures to be used in case of implementation of new interpretative nutrition labelling:<br>• Creation of a monitoring system/network group for nutritional literacy and health indicators, adherence by industry and by consumers<br>• Retrospective HIA (3 to 5 years post implementation)
**How will the HIA budget be spent?**<br>Consider:<br>• Human resources<br>• Venue hire, catering and travel costs for meetings and workshops<br>• Costs associated with dissemination of the results<br>• Evaluation costs	Human resources (project team) and logistics of the focus groups, interviews and survey (including phone calls, coffee breaks, gift cards and travel costs)

**Operating arrangements for the steering group including:**<br>Chair<br>Date and location of meetings<br>Secretariat	Meetings will take place in January and February 2019, and will be chaired by Pedro Graça, being held in Directorate-General of Health

References

[1] Cowburn G, Stockley L. Consumer understanding and use of nutrition labelling: a systematic review. *Public Health Nutr* 2005; 8: 21–28.

[2] Macera CA. *Promoting healthy eating and physical activity for a healthier nation*, http://www.cdc.gov/healthyyouth/publications/pdf/pp-ch7.pdf (2003).

[3] WHO Regional Office for Europe. *Health impact assessment. Main concepts and suggested approach*. Brussels, http://www.healthedpartners.org/ceu/hia/hia01/01_02_gothenburg_paper_on_hia_1999.pdf (1999).

[4] Institute of Public Health in Ireland. *Health Impact Assessment Guidance*. The Institute of Public Health in Ireland: Dublin, 2009.

Publisher's Note: MDPI stays neutral with regard to jurisdictional claims in published maps and institutional affiliations.

© 2021 by the author. Licensee MDPI, Basel, Switzerland. This article is an open access article distributed under the terms and conditions of the Creative Commons Attribution (CC BY) license (http://creativecommons.org/licenses/by/4.0/).
